# Fostering Children’s Connection to Nature Through Authentic Situations: The Case of Saving Salamanders at School

**DOI:** 10.3389/fpsyg.2018.00928

**Published:** 2018-06-08

**Authors:** Stephan Barthel, Sophie Belton, Christopher M. Raymond, Matteo Giusti

**Affiliations:** ^1^Faculty of Engineering and Sustainable Development, University of Gävle, Gävle, Gävle; ^2^Stockholm Resilience Centre, Stockholm University, Stockholm, Stockholm; ^3^Department of Landscape Architecture, Planning and Management, Swedish University of Agricultural Sciences, Alnarp, Alnarp

**Keywords:** nature experience, affordances, affective relationships with nature, urban, situated learning, stewardship, qualitative methods, longitudinal approach

## Abstract

The aim of this paper is to explore how children learn to form new relationships with nature. It draws on a longitudinal case study of children participating in a stewardship project involving the conservation of salamanders during the school day in Stockholm, Sweden. The qualitative method includes two waves of data collection: when a group of 10-year-old children participated in the project (2015) and 2 years after they participated (2017). We conducted 49 interviews with children as well as using participant observations and questionnaires. We found indications that children developed sympathy for salamanders and increased concern and care for nature, and that such relationships persisted 2 years after participation. Our rich qualitative data suggest that whole situations of sufficient unpredictability triggering free exploration of the area, direct sensory contact and significant experiences of interacting with a species were important for children’s development of *affective relationships* with the salamander species and with nature in an open-ended sense. Saving the lives of trapped animals enabled direct sensory interaction, feedback, increased understanding, and development of new skills for dynamically exploring further ways of saving species in an interactive process experienced as deeply meaningful, enjoyable and connecting. The behavioral setting instilled a sense of pride and commitment, and the high degree of responsibility given to the children while exploring the habitat during authentic situations enriched children’s enjoyment. The study has implications for the design of education programs that aim to connect children with nature and for a child-sensitive urban policy that supports authentic nature situations in close spatial proximity to preschools and schools.

## Introduction

Globally, the number of urban dwellers is projected to increase from 3.2 billion in 2005 to about 6.4 billion by 2050 (UN-Habitat, 2016). This unpreceded rate and scale of urbanization may embark civilization on a development trajectory with limited possibilities for people, and especially for children, to experience natural environments on a regular basis (Giusti, 2016; Colding and Barthel, 2017; Hand et al., 2017). Such a development trajectory, if not carefully designed, may lead to a shift in baseline related to connection to nature (Miller, 2005; Giusti, 2016; Hartig and Kahn, 2016; Soga and Gaston, 2016), defined as ‘one’s affective, experiential relationship to the natural world’ (Mayer and Frantz, 2004, p. 504).

Multiple studies in distinct scientific traditions have considered adults’ connectedness to nature, otherwise termed human-nature connections, and its relationship to pro-environmental behavior (e.g., Raymond et al., 2010; Brehm et al., 2013; Restall and Conrad, 2015; Ives et al., 2017). In the environmental psychology literature, connectedness with nature can be seen as a psychological construct that reflects the degree to which individuals perceive that they are part of the natural environment (Schultz, 2001, 2002), and such perception arguably influences adults’ motivations to engage in pro-environmental behavior (Kals et al., 1999; Chawla, 2007; Ernst and Theimer, 2011; Cheng and Monroe, 2012; Liefländer et al., 2013; Mayer and Frantz, 2014; Richardson et al., 2016). For example, environmental activists report greater connectedness with nature compared to college students, suggesting that connectedness with nature may be related to pro-environmental attitudes and behaviors (Bruni and Schultz, 2010). However, recent studies indicate that the attitude–action gap is still an unresolved scientific problem in sustainability planning and management (Sörqvist, 2016; Kaaronen, 2017; Linder et al., 2018).

Scholars have recently begun to unpack the meaning of children’s connection to nature and its association to different nature-based activities (Ernst and Theimer, 2011; Cheng and Monroe, 2012; Giusti et al., 2014, 2018; Giusti, 2016). For example, the findings of Cheng and Monroe’s research (2012) suggest that learning, understanding, and experiencing nature are all factors that can positively influence the development of a child’s affective relationship. However, the many studies aiming to evaluate an activity’s effectiveness in enhancing children’s connection to nature employ quantitative methods (usually performing pre- and post-activity tests), with results indicating no, or very little, change (Ernst and Theimer, 2011; Bruni et al., 2015; Giusti, 2016).

In response to this shortcoming, there is a need for longitudinal studies for assessing the changes in children’s connections to nature across time. Furthermore, research is needed to assess what specific aspects of environmental education programs encourage positive shifts in connection to nature (Ernst and Theimer, 2011; Kossack and Bogner, 2012; Liefländer et al., 2013).

In this paper, we focus on if and how urban children strengthen their connection to nature, used here open-endedly as an affective relationship with nature characterized by empathy for creatures, enjoyment of nature, sense of oneness, and sense of responsibility (cf. Cheng and Monroe, 2012). We focus on a case where young children take part in a stewardship project aiming to save endangered species, the Salamander Project, and how their participation shapes their connection to nature. Our focus on children is important given that people acquire strong connections to nature most easily during childhood, by participating in social action, but also by first-hand sensory interaction with natural environments and species (Kahn and Kellert, 2002; Mayer and Frantz, 2004; Chawla, 2006, 2007; Wells and Lekies, 2006; Chawla and Cushing, 2007; Evans et al., 2007; Cheng and Monroe, 2012).

The aim of this paper is to increase our understanding about *if, how* and *by which means* children’s affective relationships with nature change by taking part in a nature conservation project during school hours, and if such a shift persists 2 years post-participation. Does participating in the Salamander Project at school strengthen children’s connection to nature? If so, how do children learn to create affective relations with nature? Which specific situations might encourage or enable stronger affective relationships with nature? Do affective relations persist 2 years after the project? This paper studies fourth graders (age 10–11) who, during an 8-week period, took part in saving salamanders near their school in Stockholm. It started with a master’s thesis study (Belton, 2016), which then transformed into a longitudinal study to explore whether changes in affective relationships with nature detected in these children persisted long after their actual hands-on engagement.

### Theory on Learning, Behavior and Change

Our work is firmly rooted in evolutionary thinking and framed by a social-ecological systems approach that assumes that learning emanates from human behavior in interplay with features of both the biophysical and social environment. It views human beings like other creatures in the web of life with which they have co-evolved, claiming that people, like other organisms, encounter the physical world directly with all senses, with the ability to perceive qualities of the world that are really there rather than merely mental constructions about the world (Chawla, 2006). At the same time it does not deny the powerful influence of socialization and culture (Wenger, 1998; Clark and Uzzell, 2002). Instead the frame views socio-cultural influences to shape, rather than to prevent, how we select and use the information that we receive about the world’s true qualities. Affordance theory (Gibson, 1979; Kaaronen, 2017) and situated learning (Lave and Wenger, 1991), specifically fit well with such a social-ecological approach.

### An Affordance Approach to Environmental Behavior and Learning

Affordance theory is grounded in ecological psychology and interprets human behavior from a dynamic and coupled systems approach (Gibson, 1979). Ecological psychology is interested in environmental learning and action in every setting, and is particularly well adapted to describe what happens when children learn through autonomous movement and exploration, such as children at play outdoors (Kyttä, 2004; Chawla, 2006). An affordance, in its simplest understanding, refers to the action possibilities provided by objects or environments. However, a more nuanced understanding of affordances does not consider affordances as properties of objects or environments, but rather in terms of *whole situations* (Chemero, 2003, 2009). Affordances are defined as the ‘relations between abilities to perceive and act and features of the environment’ (Chemero, 2009, p. 150). For actualisation to occur different characteristics of the individual, such as his/her physical abilities, emotions and intentions must be matched with properties of the physical environment (Kyttä, 2004; Chemero, 2009; Roe and Aspinall, 2011; Withagen et al., 2012).

Children are most likely to stay attentive and engaged when perceiving affordances that provide them with immediate, pleasurable experiential feedback about the effects of their actions (Heft and Chawla, 2006; Beery and Jørgensen, 2016). Such feedback may be perceived if children are allowed, and are able, to shape features of the physical environment and experience with all five senses changes in their environment as a result of their actions, such as when building a dam with small rocks in a stream (Chawla, 2006), or when saving the life of an animal that is trapped. Kyttä (2004) notes that these situations are characterized by positive interactive cycles: the more widely that children move through and experience their world, the more satisfying encounters they have with engaging affordances, which motivates them to explore them even further. Even if the socio-cultural aspects of the environment always have been present in affordance theory (Gibson, 1979), it has of late been brought more to the foreground (Raymond et al., 2017). Affordances are dynamic and coupled human-environment relations (Kaaronen, 2017), where behavior is probabilistic and actualisation of affordances may occur when the right social circumstances are present (Clark and Uzzell, 2002; Kyttä, 2004). A situated learning approach is used below to explore how affordances are related to connection to nature and learning about nature.

### A Situated Approach to Learning

Several well-established theories and disciplines are nested under a situated (ecological) approach to learning (Lave and Wenger, 1991; Barab and Roth, 2006; Durning and Artino, 2011). In brief, situated learning understands all learning and thinking as inevitably located in activity (in doing) and, therefore, *inseparable from experience* (Brown et al., 1989; Lave and Wenger, 1991; Barab and Roth, 2006; Bendt et al., 2013). Many of the skills required in everyday settings are learned mainly through behavior, or through practice (Lave, 1988; Saxe, 1991), whether tacit and unconscious or explicit and codified (Leonard-Barton and Sensiper, 1998). Skills are not always articulated, it is simply what we do, or ‘what changes our ability to engage in practice, the understanding of why we engage in it, and the resources we have at our disposal to do so’ (Wenger, 1998, p. 97). If the concept of culture can be defined as a system that gives meaning and significance (Geertz, 1993), learning from a situated view is innately connected to the production of identity and meaning (Lawrence, 2009).

Learning can therefore be viewed as a product of people’s enculturation, in turn related to the accepted norms and values of groups, within which the individual acts (such as a stewardship group) (Lave and Wenger, 1991; Wenger, 1998, 2000). If environmental values of a participator are also reflected in the shared norms of the group, pro-environmental behavior is supported by positive feedback mechanisms provided by other group members, for instance, by way of comments, bodily postures or rewards. The social norms of the group form part of the individual member’s choice architecture, in combination with properties of the physical environment. The more authentic a situation is experienced to be (i.e., the closer the situation is to resembling real life), the better a learning opportunity it provides (Reed, 1996; Boyer and Roth, 2006; Durning and Artino, 2011). As far as ‘real life’ situations go, scholars of environmental education and child development point to nature experiences as being particularly rich in opportunities for meaningful learning activities (Kyttä, 2002; Chawla, 2007; Moore, 2014; Beery and Jørgensen, 2016). This theory increasingly acknowledges socio-material features to shape situations for learning (Godden and Baddeley, 1975; Smith and Vela, 2001; Barab and Roth, 2006; Muro and Jeffrey, 2008). Hence, urban environmental education is one means by which children actualize the affordances offered by nature in cities (Chawla, 2006; Delia and Krasny, 2018).

### The Salamander Project

A pond in Olovslunds Park in Bromma (a suburb of Stockholm) is one of the most important breeding habitats within the greater Stockholm region for the two species of salamander found in Sweden^1^, the common newt (*Lissotriton vulgaris*) and the great crested newt (*Triturus cristatus*)^∗^. The pond, being shallow, relatively warm and free from aquatic predators, provides an ideal habitat for these amphibians to reproduce. However, salamanders often fall in a concrete wading pool (located adjacent to the pond) during their annual migration to spawn in the pond in spring (**Figure 2**). The salamanders are unable to escape the wading pool because it is drained of water at that time of the year, and therefore dry out and die.

**FIGURE 1:**
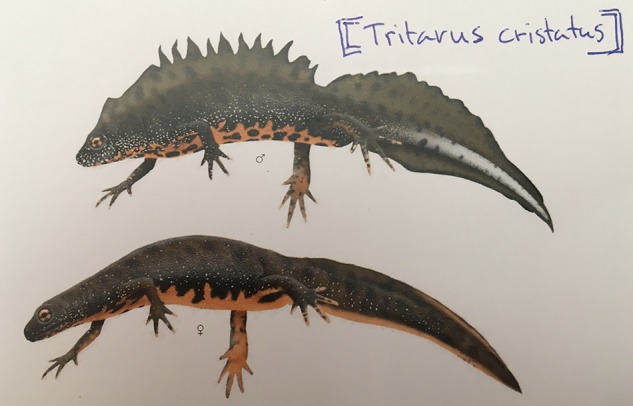
The protected great crested newt (*Triturus cristatus*). Photo of postcard, Source: ArtDatabanken.

In 2007 the local authorities, realizing how serious an issue this was, developed a pond management plan. Both salamander species are protected under national law (Länsstyrelsen, 2015) and the great crested newt (**Figure 1**) is listed in both the Bern Convention and Annex IV of the EU Habitats Directive requiring a ‘strict protection regime’ by member states (Lundberg and Kiibus, 2014; European Commission, 2015). Under the pond management plan, a number of technical strategies were implemented in an attempt to solve the trap problem. These, however, were not sufficiently effective, leading the local authorities to ask for help from a nearby school in 2008 (Kiibus, 2011). Bromma, where the school is located and the project takes place, is 8 km west of Stockholm. This predominantly middle to high-income residential area has a population of 70,000 (Belton, 2016).

**FIGURE 2:**
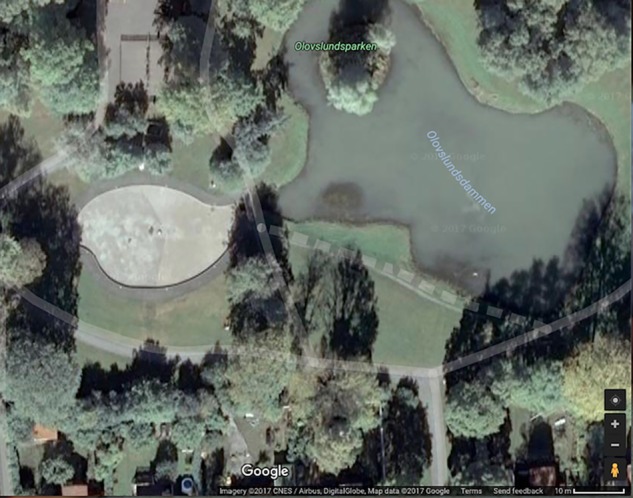
Olovslund Park with the pond on the right and the paddling pool to its left. The school is just a short walk away (source: Google Maps).

Every year since 2008, the fourth grade students of Olovslund School participate in the Salamander Project (60–70 students yearly). The aim is to save salamanders and simultaneously to teach the students about conservation issues. Every school lunch break of the salamander breeding season (April/May), a small group of children carefully search through piles of leaves scattered around the wading pool with sticks and place any salamanders found in a bucket of water. The piles of leaves are placed inside the concrete wading pool with its sharp edges to provide habitat for the salamanders to hide and escape the sun, but they ultimately dry out, or are cleared as the pool season starts, making the pool a kind of death trap. Before releasing them into the nearby pond, the children document the number, species and sex of the salamanders found, as well as from which pile of leaves each was found. These daily reports are then communicated by the teacher to a biologist enabling the tracking of migration trends, the number of salamanders trapped and saved and monitoring the effectiveness of the project.

Over the 10 years that the project has been running, 1204 great crested newts and 3715 common newts have been rescued by Olovslund School (personal communication, 2017), allowing the local salamander population to remain stable in a time when urbanization and habitat loss pose a threat to many urban amphibian species (Lundberg and Kiibus, 2014). Another measure of success from an ecological perspective is the successful re-introduction of the great crested newt species from this pond into a nearby pond in 2009, leading to the set-up of an identical operation in 2015 (Kiibus, 2011; Lundberg and Kiibus, 2014). This meant an extra task for the participants: taking the ‘saved’ great crested newts back to school (instead of releasing them into the pond). In order to do this, the school was granted special permission by the county authorities (Länsstyrelsen, 2015), it otherwise being illegal to collect this species in Sweden.

## Method: Qualitative and Longitudinal Research Design

Given the ample room for improving theory and understanding about children’s connection to nature over time, we decided upon a qualitative and longitudinal research design (Beery and Wolf-Watz, 2014; Chawla, 2015; Beery and Jørgensen, 2016). Qualitative data was collated through semi-structured interview questions and open-ended questions included in a questionnaire administered to the school children. Our attempt was to elicit experiences of the participants both by listening to, and analyzing, their own words, which helped us gain a more in-depth, ‘inside’ understanding of the *how* and *why* lines of inquiry (Patton, 2002; Yin, 2009). We also consistently observed their behavior and activities in the field.

The theoretical starting point for this study was Chawla’s (1998, 2006) framework on children’s development of affective relationships with nature. This framework includes how humans acquire affective relationships with nature most easily during childhood in situations that trigger behavior and immediate feedback, which are experienced to be of significance. In recognition that connection to nature is still a contested concept in the environmental psychology and environmental management literatures (Ives et al., 2017), we explore multiple facets of affective relationships with nature and its change over time. To support a rich and deep understanding of aspects of affective relationships with nature, we sought in part for themes to emerge from the open-ended interview and survey responses. The interview script consisted of sections that sought to explore how such relationships may be shaped by aspects both of the biophysical environment and also by the social context. Follow-up open-ended and ordinal level survey questions on the same topic enabled these patterns to be examined in further depth.

The interviews and questionnaires were administered across two waves of data collection conducted 2 years apart (2015 and 2017) in order to study if changes in children’s affective relationships with nature persisted 2 years after participation in the Salamander Project (see **Table 1**). In accordance with triangulation in qualitative research, we employ a range of data sources to explore and explain themes that cut across several sources (Creswell, 2014).

**Table 1:** Temporal phases of the empirical field study.

	Month	April – May	Early June	Late June
Wave 1 2015	Method	**Field observations** of the children participating in the project, classroom lesson and Salamander Evening (9)	**Questionnaire** Mostly open-ended questions about participants’ views on the project, post-participation (*n* = 57)	**Face to face semi-structured individual interviews** With children just after participating in the project (*n* = 25)

	**Month**	**March**	**May**	

Wave 2 2017	Method	**Face to face semi-structured individual interviews** With children 2 years after participating in the project (*n* = 24)	**Questionnaire** Mostly open-ended questions about participants’ memories and views of the project 2 years after participation (*n* = 49)	

*Two waves of data collection 2 years apart (2015 and 2017)*.

Contact with the school had been established prior to this study in the form of a pilot study in 2014. Alongside consent from the school principal and concerned teachers, full written parental consent was obtained for all participating students. Furthermore, the research design was approved in an ethical review process conducted by a Swedish university.

### Data Collection Wave One – Spring 2015

#### Field Observations of the Salamander Project

During the first round of data collection, nine field observations of the conservation project took place over a 2-month period (Appendix A). The first of these was of an initial information lesson given in the classroom by the teacher in charge of the project, explaining and preparing the students for their involvement in the project.

Seven field observations involved meeting the group of children and the teacher at the school and walking with them to the park to observe them partake in the project (about 45 min each time). These observations were spread out over the length of the project in order to capture differences across time and in differing conditions (such as weather and number of salamanders found). The same groups of children were observed at different points in time enabling us to note changes between children’s first, second, and third (final) participation. While the first of these observations was a general observation of the learning environment, the following were participant observations allowing us to experience the hands-on work, mingle with the children and ask informal questions. This participant observation was helpful because it allowed the children to become familiar with us before being interviewed. Detailed notes were taken after each observation in order to have a written record to refer back to.

Lastly, we attended the local ‘Salamander Evening’ as participant observers (see **Table 1**). This is an annual community event at the pond in Olovslunds Park where Stockholm biologists give an informal talk about the project, thank the school for their work, and proceed, with the help of all people present (children and adults), to count the salamanders in the pond.

The general aim of these observations was to witness the unfolding of the conservation project in its fullness to better understand what it embodied from the point of view of the participants (DeWalt and DeWalt, 2011). We took note of what the children did, how they did it (e.g., with what level of engagement/concentration and what type of body language), what they talked about while participating, what the atmosphere was, and how these attributes changed over time. The observations acted as springboards for developing interview questions as well as a means to verify themes and/or insights that emerged from interview and questionnaire data (Patton, 2002).

### Salamander Project Questionnaire

Students completed a short questionnaire after completion of the Salamander Project (June 2015) during regular class time (see **Table 1**). Questions were designed to mostly collect information on nominal level data (e.g., ‘Pick three words that best describe the Salamander project for you’), mixed with certain questions of more ‘closed’ character (e.g., ‘How many salamanders did you find in total?’) (Appendix B). As mentioned above, questionnaires enabled a larger sample size for us than the interviews (*n* = 57 vs. 25 in 2015 and *n* = 49 vs. 24 in 2017), which was helpful in determining whether emergent interview patterns were consistent across data sources.

### Semi-Structured Face-to-Face Interviews

During the final days of the project, 25 children who had participated in the project were interviewed (see **Table 1**). Selection criteria for the interviews (Appendix C) were based on: (1) full parental consent to interview, record and use their child’s quotes; (2) an even spread of students across the three classes so as to account for the possibility of a teacher’s pedagogical influence on children’s views about nature/the project; (3) equal gender representation; and (4) a variety in the number of times children had participated in the project (two to five times).

The purpose of the interviews was to uncover the experience of participating in the project from the children’s perspective, and to understand whether it strengthened their affective relationships with nature. The focus was on how it felt to be part of the project, what changes (if any) they had experienced during the course of the project (changes in feelings toward salamanders and nature as well as changes in themselves) and what they had learnt from it. Interviews were conducted in an informal manner in a comfortable setting (one-on-one, in Swedish, at school during school time) and, given the age of the participants, were kept short (10 min). Interviews were semi-structured following an interview guide (Appendix D), at the same time allowing the conversation to follow its natural course and for new questions to arise spontaneously (Kvale, 1996; Patton, 2002).

### Data Collection Wave 2 – Spring 2017

The second wave of data collection occurred exactly 2 years later, in spring 2017, when the participants were in sixth grade and their final months of Olovslund School (see **Table 1**). This round of data collection consisted in 24 interviews (of the same nature as in 2015: short, one-on-one, at school and during school time, see Appendix F) and a questionnaire. The focus for both the interviews and questionnaire was on: (1) what the children remembered/retained and what they had learnt from their participation in the Salamander project in 2015; (2) whether their view of salamanders, other animals and nature had changed/shifted with the project and if so, how; and (3) whether or not they feel their connection to nature had changed with their time in the project and, if so, how.

The questionnaire was a combination of open-ended and box-checking questions (Appendix E). The 24 interviewees were selected evenly across the three classrooms, and were a mixture of children that had previously been interviewed in 2015 and those that hadn’t (Appendix C). Hence, a mixture of children researched on both waves of data collection and those only partaking 1 year formed part of our unit of analysis. This unit was chosen because we wanted our unit of analysis to represent a fair gender balance and simply for practical reasons (voluntary to participate), and also to explore a wide range of views. In 2017 no observations took place, as the participants did not take part in the Salamander Project (a fourth-grade activity only).

### Analysis of Data

Interviews from both waves of data collections were transcribed verbatim and coded for emerging themes, using the software program Dedoose (version 6.2.17). Quotes were then translated into English. Coding was done in two ways. Firstly, in terms of exploring qualitative aspects of affective relationships with nature, we coded the interview data for three of the four sub-constructs of connection to nature as developed by Cheng and Monroe (2012): enjoyment of nature, empathy for creatures and sense of responsibility. The fourth sub-construct (sense of oneness) did not work well for this age group in our opinion.

Secondly, we were interested in exploring the conservation project’s specific features that appeared to have facilitated the development of affective relationships. Here, coding was done in terms of emergent themes that surface from analysis of the interview data—an iterative process that required several rounds of analysis. The written texts from the children in the questionnaire data were first translated into English and then analyzed looking for recurring themes with the help of the software program NVivo (version 11) and the website Woodle. Themes that emerged from any of the data sources were compared with our other data sources whenever feasible. For example, interview codes were considered when analyzing questionnaire data and vice versa, leading to code/theme refining. Additional gray and scientific literature, informal written and verbal conversations with the children’s teachers, the local biologist and the teacher in charge of the project, were equally analyzed in light of themes that emerged from primary data.

## Results

Interview and questionnaire results from 2015 point to a self-observed change in children’s connection to nature after project participation. This change was described as a positive one: increased concern, interest in and/or care for nature. Answering interview questions about how they had changed with the project, 16 children (out of 25) expressed increased empathy toward salamanders (‘feeling’ and ‘caring’ more for them). They explained that they had developed a better understanding for salamanders, both in terms of facts about them (e.g., how to differentiate amongst species and sex), but also how to ‘help’ and ‘care for’ them. An emergent finding from our empirical material was that 17 children (out of 25) noted that they had learnt more. Furthermore, in the questionnaire, 93% of children answered ‘yes’ to caring more about salamanders after the project than before. When talking about changes in how they felt toward salamanders and changes in themselves many children talked about going from not knowing or caring much about these amphibians to being closer to them, much more aware of them and thinking more about them:

*I have more — okay, respect is a big word but I have to use it because there isn’t another one — respect for how they [salamanders] live because it’s quite… I wouldn’t survive if I were a salamander!… Now I see them in a different way. Before I thought they were like animals. Now it’s like they are beings that, well, they need help, just like people can need help sometimes*.
(Participant 1)
*We got to know them*.
(Participant 11)

Shifting experiences of touching the species in question was an emergent theme from the interviews. In time, several children got over their initial fear of touching salamanders. Eight children experienced a change from being scared of, or nervous about salamanders, or finding them ‘creepy,’ to feeling more comfortable with them and daring to hold them as this following quote depicts:

Interviewer: *Do you think that you have changed with the project?*
Participant 4: *Umm… before, I was a bit scared of salamanders. They were, like, a bit slimy. I didn’t dare to hold one and now I can hold one without any problem… I have, like, stopped being scared of them*.

This increased ‘connection’ to salamanders was mirrored by strengthened relationships with nature in a broader, more open-ended sense. Children typically expressed thinking and caring more about other animals as well as about nature (in the wider meaning of the concept) after the project. The following quotes exemplify common answers to the question ‘Do you think you have changed with the project?’

*I have learnt to take care of animals. I’m maybe thinking about doing something like that maybe… to fix things so that everything is good with nature.… Yes… I have become more nature-friendly*.
(Participant 6)

*Yes, well, I have much more of a sense for nature and salamanders*.
(Participant 3)

*That’s a hard question!... Well, I have started to think more about animals and nature. Actually a lot more than what I did before*.
(Participant 25)

*Well, it’s like I’m less scared and I feel more... confident in nature*.
(Participant 23)

The main result from 2017 shows that the affective relationships that formed in 2015 remain. When asked directly if they cared about salamanders and if they would help a salamander if they saw someone hurting it, all interviewees answered ‘yes.’ The vast majority of children expressed that their feelings toward salamanders had changed with the project, reflected in an increased empathy toward salamanders (83% of interviewees), as the following quote from 2017 expresses:

Interviewer: *Did your feelings for salamanders change with your time in the project?*
Participant 23: *Yes they did actually. I hardly knew what a salamander was before the project so… they have. I know what a salamander is now and I care more about them!*

Even as 12 or 13-year-olds, in 2017, the majority of children (71%) expressed that the Salamander Project had changed the way they view other animals: they think and care more about them as well. Also, the majority of children (75% of interviewees, 54% of questionnaire respondents) confirmed that their view of nature (in the broader sense) had changed with the Salamander Project: it had helped them understand how important nature is and particularly how important it is for animals. Some children had more place-connected answers, thinking more of nature as a home for salamanders since the project, or explaining that their view of nature as a whole hadn’t changed, but that their view of the specific salamander habitat had.

As the findings above show, the Salamander Project has helped children relate to salamanders but also to other animals (albeit to a lesser extent) and even to nature (in a more theoretical, less concrete way). This is further confirmed by the answers to the question ‘Do you feel you, as a person, have changed with the Salamander Project, and, if so, how?’ Out of the 79% of interviewees who answered yes, the vast majority of answers were salamander-specific (i.e., I care more about salamanders now), followed by animal-specific answers (i.e., I think more about animals now), and lastly answers that were about nature or something broad (i.e., I am more careful now, I know more about nature now). Indicators of shared memories include that 75% of interviewees in 2017 had thought about salamanders or the project, and 83% had talked about salamanders or the project during the last 2 years. When asked to recount their memories, interviewees spoke fondly about the project and 83% thought that they would remember it when they are older. An indicator that emerged from our empirical material of increased sensitivity toward salamanders is that 91% of interviewees had observed salamanders in the nearby surroundings during the 2 years that had passed.

### Participating in Something of Significance With a Sense of Responsibility

Children highly valued being ‘part of’ the project, being given the opportunity to ‘participate’ in or be ‘included’ in an important real-world project. For example, the contributing/helping aspect of the project (the word ‘saving’ was recurrent in both interviews and questionnaires) emerged as important for the children not only in terms of physically helping salamanders, but also with regards to having a role in the wider community by helping the municipality with its duty of biodiversity protection. This finding is supported by situated learning that stresses the importance of goal-driven activity for meaningful learning (Chawla, 2006; Durning and Artino, 2011).

Participant 21: *I think it’s really nice that we can help out…. The teacher explained that… only our school has permission to take the great crested newts and I think that that’s pretty cool*.
Interviewer*: It is!*
Participant 21: *And we can talk about it later when we are big, to our children*.

**FIGURE 3:**
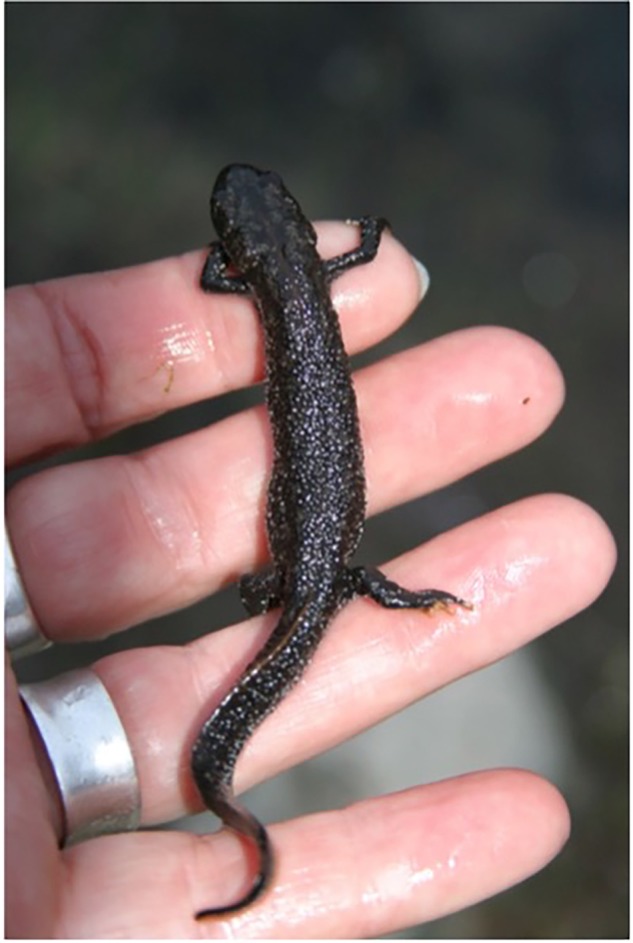
Developing affective relationships with nature by way of significant experiences when saving the life of an animal, here by handling the species: carrying a great crested newt in one’s hand. Photo: Martina Kiibus.

Being part of a ‘bigger’ project and community context (participating in an adult activity, beyond school) gave children a sense of importance, responsibility and pride. Children clearly expressed their sense of responsibility toward the Salamander Project and their appreciation for being given this responsibility. This was apparent throughout the interviews but was also recognized by the teachers who noticed how committed their students were to the project and the sense of importance that stemmed from it. This was further confirmed by observations in the field of children’s careful concentration and thoroughness in the tasks (**Figure 3**).

Children talked about how things changed over the course of the project and how they had an increasing amount of responsibility, as the teacher trusted them to do the job well. One student called this ‘freedom with responsibility’ (Participant 25). This increased responsibility, trust and freedom allowed the children to get closer to and be sensitized to the salamanders in their own time and manner, and also to gain increased skills by repeatedly performing the tasks.

*Well it was nice that she [the teacher] didn’t come and watch over us, rather she thought we could do it and we could! We had to take responsibility but it was, like, fun to have it*.
(Participant 9)

Results from 2017, show, just like in 2015, that children valued ‘participating in,’ ‘being part of,’ and contributing to an activity they felt was useful and important. Having responsibility in a bigger-than-school project was still seen in many instances as something special or different, as exemplified by the following quote from 2017:

*It felt important. It felt like we were helping out and that it was important*.
(Participant 30)

### The Project Provided Authentic Situations

An emergent finding was the importance of the fact that the project was a real, on-the-ground conservation project. That was highly appreciated by the children and the teacher in charge also reported that the children appreciated connecting with living organisms during authentic situations. Had it been set up as a normal ‘school’ project, she explained, they would have made sure to place salamanders in the wading pool daily to be found by the students. Instead, being an authentic project, located within the salamander migration corridor, one never knew what one would find and while most days some salamanders were found, (the record find was 70 although the usual find was less than 10), there were also days when none were found (Appendix A).

We found two specific features that illustrate how the Salamander Project provided authentic situations: its complex nature and the variability of conditions. Firstly, the Salamander Project was established as a social-ecological solution to the issue of a protected species dying because of a socially valued wading pool. The pool was the ‘death-trap’ located inside the natural breading ground of the salamanders, but this ‘trap’ also afforded the conservation of native species by children. Although a more effective strategy may have been to remove the pool entirely, authorities were unwilling to do so due to its high recreational and aesthetic value, resulting in the current solution and its reliance on Olovslund School. This situation also provided the children with a rich opportunity to reflect on the pros and cons of this arrangement. When asked what their preferred solution to the paddling pool ‘trap’ would be, their answers typically conveyed a clear understanding of the complexity of the issue:

*Well, on the one hand it would be good to take it away so that no salamanders could fall in it at night but it is still pretty fun to play in the wading pool in the summer. On the one hand, it would be good to remove it but on the other hand it’s really fun for us to keep the project up, that we, Olovslund School, can save salamanders*.
(Participant 9)

Secondly, the different and varying conditions of each participation in the project (e.g., number of salamanders found, weather, group dynamics, unexpected events) expanded opportunities for reflection and meaning-making. This finding was first identified from field observations and then followed up in interview questions. An example of this was when the children found a dead headless salamander. This allowed them to think about what could have happened to it and, with the help of the teacher, brainstorm which animals prey on salamanders. Another example is of finding a juvenile salamander in the pool. This allowed the children to see for themselves how it is impossible to identify the sex of a juvenile and provided the teacher with a good opportunity to explain salamanders’ lifecycle. These kinds of unique events, with each occurrence depending entirely on the particular conditions of the day, allowed for spontaneous opportunities to enrich children’s overall experience of the project.

Experiencing different and varying conditions was enabled by the projects’ length (2 months), which may have influenced how the children became familiar with the salamanders and the tasks involved in the project. Indeed, when asked about the differences between the first and last time in the project, 15 children (out of 25) described how they had gotten more used to the project, felt more confident in the tasks, and knew more about the scope of the project as well as about salamanders.

The 2017 interviews revealed, again, the extent to which the children recalled the project’s authentic situations, as something very special. Students talked enthusiastically about moments during the project that were unusual or surprising such as finding a baby salamander or a dead salamander, or a particular time when very many salamanders were found. These types of moments were made possible due to the ever-changing conditions provided by the conservation project, which also seemed to have enabled enjoyable and exciting experiences.

### Fun and Excitement

All but one of the children interviewed in 2015 considered the project ‘fun’ and readily expressed the enjoyment they got from it. The 2015 questionnaire showed that ‘fun’ was chosen by 66% of children as one of the three words that best described the project for them. Although the project took place during lunch break (meaning that children skipped their usual break activities), 91% of questionnaire respondents said that it didn’t feel like they had lost a break. Field observations and informal conversations with the students’ teachers supported interview and questionnaire results. The teachers noted the joy and enthusiasm their students got out of the project, particularly evident directly after their turn participating. This quote captures these findings:

*It’s fun to feel that you have done something important. Something that is actually good for the environment, something that makes a difference*.
(Participant 1)

Children gave several reasons for why the project was ‘fun’ of which the most common were: having responsibility, being included in something ‘big’ and the fact that the project was ‘real.’ This ‘fun’ element is therefore tightly linked to the other features described previously. The different descriptions of why the project was ‘fun’ suggest that this word has a multi-dimensional meaning for these children, encompassing many positive and complementary qualities.

In 2017, all children interviewed expressed the enjoyment that they got from the project, making this the aspect of the experience that appeared to have stuck most vividly in their memory. The 2017 questionnaire results confirmed this finding, as 94% answered that they had liked taking part, and 69% used the word ‘fun’ to describe their experiences. An emergent finding was that ‘helping’ was the most common reason for why children enjoyed the project (51%). It was fun to help/save salamanders (the most common answer) but also to ‘help out,’ to help animals and to help nature. The second most common explanation for it being fun was because it was interesting (20.4%).

### Self-Reflection on Methodological Approach and Results

Our methodological approach has some important limitations that need to be mentioned. One significant study limitation relates to the challenge of children age 10 or 11 having to translate their experiences into words. We understand if readers cannot ascertain whether our findings are related more to changes in skills in for instance handling of salamanders, or to changes in deep seated *emotional components* of connection to nature (Kals et al., 1999; Cheng and Monroe, 2012). To mitigate this methodological weakness, we tried to verify patterns that emerged from interview data by using (1) observations of behavior in the field; (2) questionnaires; and (3) interviewing the children 2 years later when their verbal skills are more developed (see **Table 1**). Participant observations detected change in children’s *feelings* toward salamanders, as much as is possible from an observation (feelings are, of course, deeply internal and personal and cannot be conveyed entirely ‘outwardly’ through, for example, actions, facial expressions, body language or verbally). Through examining these characteristics, however, we did detect that children (1) became more comfortable with handling salamanders (some children went from being scared of them and finding them ‘yucky’ to being unafraid and finding them ‘normal’ or even ‘sweet’); and (2) showed more interest in and affection toward them. The questionnaire results pointed to a self-observed positive change in children’s *feelings* toward (as well as knowledge of) both salamanders and nature. Patterns from the first wave of interviews were also apparent in the second wave of interviews 2 years later. In fact, we observed that, in 2017, the children (now 12 or 13 years old) expressed themselves in a considerably more concise way and with greater ability to discuss and reflect upon their experience in the project, which reaffirms our findings and increases their credibility.

Here we considered respondents from both waves of data collection, and those that were interviewed only one of the years as the same unit of analysis. We analyzed differences in views and meanings in the empirical material and no deviating patterns were found in responses from children that were studied during both 2015 and 2017, and those studied only in 2017. We acknowledge, however, that not restricting the unit of analysis to participants that took part in both waves of data collection limits our evidence concerning changes in affective relationships with nature at the individual level, compared to a strict ‘within person research design.’ We also acknowledge that ample room exists for developing better tools and methods for gathering data both on learning as embodied in, and produced through, practice and to capture changes in affective relationships in this age group. Furthermore, the qualitative nature of the study, in combination with the limited number of children available for investigation, means that our findings are not deemed general beyond the context of this study, which is why we phrase our results as indicative rather than conclusive.

## Discussion

The main finding is that learning through taking part in a local species conservation project (the Salamander Project) during school hours was associated with strengthened connection to nature and, specifically, to the salamander species. We observed a self-reported new and personal meaning for the children who expressed both increased empathy for the species in question and an increased concern for nature. These findings are consistent with previous work (Louv, 2005; Chawla, 2006; Beery and Jørgensen, 2016). However, we build on existing theory by demonstrating that these new affective relationships with nature can persist over time. The main result from 2017 shows that the strengthened affective relations observed in 2015 remain, as represented by the self-reported change in connection to nature. This shift was more discernible in 2017 when the participants were 12–13 years of age: they showed deeper reflection and talked more about the consequences of their actions, compared with when they were 10 or 11 years old.

We have no causal evidence, and there may be a plurality of explanations for why such a shift persisted (Bogner, 1998; Liddicoat and Krasny, 2013), but previous literature has showed that the developmental phase of the children likely played a part in enabling a shift in affective relationships with nature (e.g., Liefländer and Bogner, 2018). Sobel (1993) argues that the ages between 6 and 11 are particularly important for children to form relationships with nature, and for understanding themselves in relation to nature. Liefländer and Bogner (2014) found that children older than 11 years of age tend to experience nature mainly through social relations, whereas children younger than 11 seem to experience nature mainly through exploration and direct sensory contact. Indeed, the fourth graders in this study were at a stage in their developmental process where they also began to experience the natural world indirectly by participation in social action. For example, in 2017 the children showed signs of being closer to puberty and more tuned into ‘the social.’

In the following sections, we discuss our findings with reference to situated learning and affordance theories. Such a theoretical frame is used when responding to the *how* and *why* lines of inquiry in this manuscript. We further relate our insights with challenges in urban sustainability. For this we use three sub-headings:

*Exploration and experience under authentic situations; The culture of the behavioral setting; Counteracting broad-based processes toward weaker connection to nature*.

### Exploration and Experience Under Authentic Situations

**FIGURE 4:**
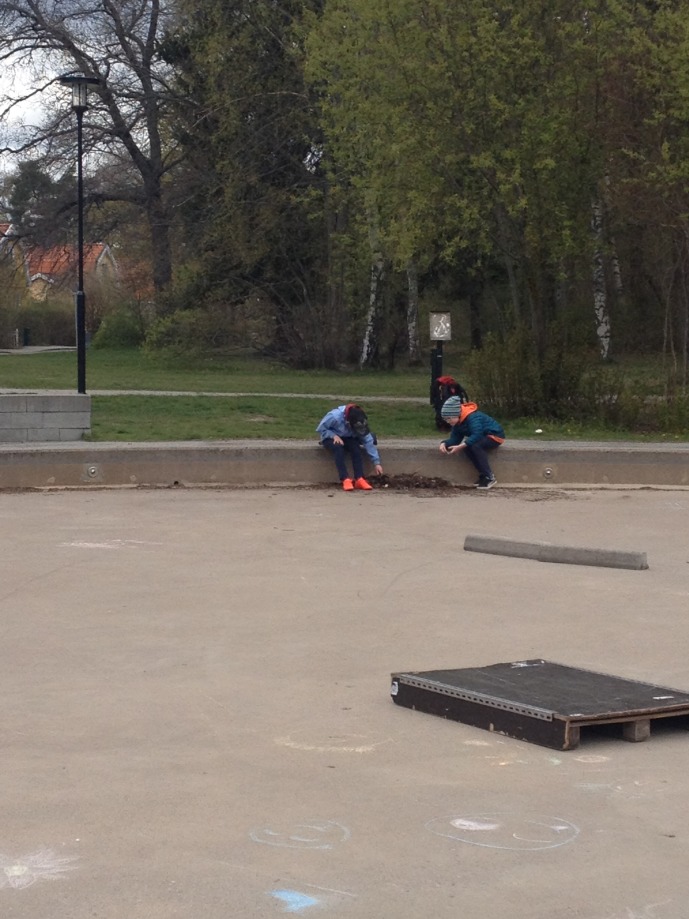
Illustration of the authentic situation of the Salamander Project. Fourth graders searching for salamanders in a pile of leaves in the wading pool during the lunch break on a chilly spring day. Consent received from parents of the children. Photo: Sophie Belton.

We interpret from our data a dynamic development of increased competence in stewardship actions. The behavioral setting of the Salamander Project enabled the children to move around relatively freely (crawl, walk, and run) and explore the salamander habitat, which we theoretically link to interactive cycles of learning-by-doing (cf. Kyttä, 2004). Such interactive cycles have been characterized by a safe world for learning constituted of responsive affordances and graduated challenges that children learn to master. As children move around and explore affordances and features of the environment, and as they overcome challenges in their environment, they build environmental sensitivity and at the same time personal competence (Kyttä, 2004; Chawla, 2006; Beery and Jørgensen, 2016). We observed positive interactive cycles of exploring the area while developing abilities over time. Under shifting situations, children were searching in the piles of leaves with a stick; finding, discovering, holding the animals in their hands; carrying and releasing salamanders into the nearby pond, and watering the piles of leaves before leaving the park, so that the salamanders falling into the pool during the night would not dry out (**Figure 4**).

Interpreted through the lens of situated learning, such learning-by-doing activities are inseparable from experience (Brown et al., 1989; Lave and Wenger, 1991; Barab and Roth, 2006). Significant sensory experiences observed include overcoming initial feelings of disgust for salamanders, shifting to emotions of empathy. Experiences of taking part in something important, and feelings of responsibility, pride and having fun were also reported. Significant sensory experiences have been identified as important transformative moments since these may create long-term effects on environmental commitment (Chawla, 2001, 2007). In environmental education, it is argued that children’s connection to nature can become strengthened as a complex ability that can come about through a series of situations that generate significant sensory experiences (Chawla, 1998; Beery and Jørgensen, 2016; Giusti et al., 2018).

Unlike a typical school activity, the Salamander Project afforded first-hand sensory explorations during authentic situations. Reflective, emotional and physiological experiences were intertwined with learning about salamanders as a species, their behavior, their habitat requirements, and how to personally relate to them. Therefore, the uncertainty inherent in the project may have been an important factor that engaged children to create stronger affective relationships with nature (Chawla et al., 2014; Moore, 2014). Moore (2014) calls the variation in environmental factors ‘sufficient unpredictability’ and understands this as important in maintaining a child’s fascination and attention (see also Chawla et al., 2014). The Salamander Project provides a good example of its unpredictable nature upholding a child’s excitement for, and interest in, stewardship practices.

Our rich qualitative data suggest that whole situations of sufficient unpredictability, triggering free exploration of the area, direct sensory contact and significant experiences of interacting with species, had a role in enabling children’s development of *affective relationships* with the salamander species and with nature in an open-ended sense. The sensory interaction was observed to enrich the children’s immediate experiential feedback about the effects of their actions. Heft and Chawla (2006) report that such feedback may be perceived if children are allowed, and are able, to shape features of the physical environment and experience with all five senses changes in their environment as a result of their actions. Here saving the lives of trapped animals gave them such feedback, which in turned seemed to have triggered interactive cycles of: direct sensory interaction, experiential feedback about the effects of actions, increased understanding, and development of new skills for dynamically exploring further ways of saving species. These observations support previous work on links between learning and sensory experiences in nature-based activity (Kyttä, 2002; Chawla, 2007; Chawla et al., 2014; Moore, 2014; Giusti et al., 2014; Beery and Jørgensen, 2016).

### The Culture of the Behavioral Setting

Behavioral settings are recurring patterns of behavior in designated places where people gather to engage in particular activities at particular times (Barker, 1968). The culture of stewardship in this school played a part in teaching the children about the accepted norms and values of the project, which also seemed to have played a part in the actualisation of affordances congruent with stewardship behavior. Indeed, most students participated even though it was not compulsory to do so. The social interactions that occur within these settings are constituted by social cues upheld by the communities in question (Lave and Wenger, 1991). Any individual member of a community of practice is affected by such social structures (Wenger, 1998), which form part in the shaping of choices (Linder et al., 2018), which further may re-constitute the behavioral setting in question. Indeed, the Salamander Project has been running for 10 years and has become a part of the school’s culture and identity, embedded in the school’s routines and engaging not only the fourth graders, but also teachers. The particular school values and pride around this project, where the school logo is a salamander and the project is mentioned on its website, appear to have encouraged children’s commitment to the project.

The behavioral setting also seemed to have made pupils stay attuned during the entire 2-month period, despite the fact that the actual amount of hands-on participation time was relatively short: on average 2.25 h per child (3 × 45-min sessions). Between the hands-on engagement, they were, however, involved in social action that potentially could remind them of the project. They studied the salamander data records and took part in classroom teaching about the species and its habitat. Despite the project running over a 2-month period, the teachers continued to talk about the project regularly after lunch asking students about the daily finds. Furthermore, not all children participated at the same time (one small group of children per day), but even the days when they did not participate, friends would return to the schoolyard with their stories to tell about the ‘catch of the day.’ Such social feedback meant that interest and involvement (even if by proxy) in the project seemed to have been kept alive. Also, the Salamander Evening (a ceremony outside school hours) seemed to have functioned as a sort of ‘memory’ (Barthel et al., 2010), which potentially reminded them of their part in the project. The Salamander Evening also seemed to have played a part in instilling a sense of pride in partaking in a ‘bigger-than-school’ societal activity. Indeed, previous research suggests that experiences of intimate interaction with nature in educational behavioral settings provide children with important learning opportunities, including actions that may help shape their relations with, and knowledge about, biodiversity (Chawla, 2001; Fjørtoft, 2004; Herbert, 2008; Ernst and Tornabene, 2012; Beery and Jørgensen, 2016).

Multiple co-benefits of the project became apparent through observations and conversations with the teachers. These included: improved science learning, the spread of local species knowledge (often from child to parent), and increased interest in the wider community. In this sense, the project can be viewed as a driver of important processes within, and a component of, a larger social-ecological system. This finding is supported by resilience scholars and reinforces their view that environmental education and learning should not be viewed in isolation from building resilient social-ecological urban systems but as an integrated and necessary part of it (Krasny et al., 2010; Russ and Krasny, 2017).

### Counteracting Broad-Based Processes Toward Weaker Connection to Nature

In terms of practical policy advice, this paper shows that involving 10 and 11-year-old children in authentic stewardship actions as an activity at school, while urban planners consider potential for learning in nature environments in close proximity to schools and kindergartens, may be a promising combination for societies to counteract broad-based processes toward weaker connection to nature (Giusti et al., 2014, 2018). Obligatory stewardship projects during school hours can be implemented more broadly in a society compared to projects that are voluntary after school. Such obligatory policy may be a necessary step if urban civilisation will stay emotionally connected to the biosphere, while simultaneously fostering social health and pleasurable feedback cycles between children and nature (Chawla et al., 2014; Chawla, 2015; Carrus et al., 2015; Collado and Staats, 2016; Samuelsson et al., 2018). As the result herein is indicative, more research is needed in order to generalize whether such policy advice has the potential to function as a deep leverage point, by supporting much needed sustainability transitions of broad-based socio-cultural processes of self-concept change and social norm formation (Meadows, 2008; Westley et al., 2011; Abson et al., 2017; Görg et al., 2017).

## Conclusion

Our findings support Cheng and Monroe’s (2012) suggestion that learning, understanding, and experiencing nature are all factors that can positively influence the development of a child’s connection to nature, here operationalized as an affective connection. This paper also develops, with emphasis put on the participants’ own views and words, an understanding about how, and by which means, the participating children’s connection to nature shifted over time. The study highlights:

• The behavioral setting of the bigger-than-school project instilled a sense of pride and responsibility.
• Children’s free exploration of the habitat during situations characterized by unpredictability enriched their enjoyment.
• Contact with species triggered direct sensory feedback of actions and enabled significant experiences.
• Significant experiences when developing sympathy in the process of going from viewing species as ‘yucky’ to viewing them as ‘sweet.’
• Experiences were intertwined with learning about endangered species and their habitat.
• Sixth graders observed salamanders even 2-years after participating in the project.

The *affective relationships* that the children formed with the salamander species and with nature in an open-ended sense, seemed to have emanated from whole and authentic situations that granted the children immediate feedback about the effects of their actions when saving the lives of trapped animals. Such situations triggered positive interactive feedback cycles of direct sensory interaction, experiential feedback as well as social feedback about the effects of actions, increased understanding, and development of new skills for dynamically exploring further ways of saving species—self-reported to generate significant and fun experiences. These complex and relational dynamics between mind, body, culture and the environment have been reported elsewhere (Kyttä, 2002, 2004; Raymond et al., 2017), but not often in the context of children’s conservation of protected species inside an urban landscape.

## Ethics Statement

Ethical implications of this study were carefully considered prior to fieldwork. Although the topic is not deemed to be of a sensitive nature, the study involved children and therefore followed specific guidelines relating to researching children (Graue and Walsh, 1998; The Research Ethics Guidebook, 2014; UNICEF guidelines for interviewing children, 2014). Furthermore, an ethical review of the research project was carried out by education staff of the Stockholm Resilience Centre, Stockholm University as a requirement. The protocol was approved by them.

All subjects gave written informed consent in accordance with the Declaration of Helsinki. Firstly, background police checks were provided to all schools involved once relevant teachers and/or principals had agreed to participate in the study. Secondly, an information letter and a consent form were sent out to all 4th grade students’ and 6th grade students’ parents/caregivers. The letter explained what the study involved, the terms of student confidentiality and anonymity, as well as how the data would be handled. Consent was asked for their child’s participation in both: (1) the classroom sessions and (2) recorded interviews where quotes could be used. Fieldwork commenced only once consent forms were collected and involved only those students whose caregivers had given consent.

Participants were met at all times with respect and study methods were adapted to the specific age groups and chosen so as to be fun and engaging activities in a familiar atmosphere (their school). Before being interviewed, students were explained that their participation was fully voluntary and that they didn’t have to answers questions if they chose not to. They were encouraged to ask questions and were explained both prior to the classroom sessions and interviews that there were no ‘right’ or ‘wrong’ answers but that we were instead interested in their personal views and feelings.

## Author Contributions

SBa was the project leader and the corresponding author. SBe did most of the field-work and did together with SBa most of the writing. MG participated in some of the early field work 2015, and he contributed with discussing the theoretical view-points together with CR.

## Conflict of Interest Statement

The authors declare that the research was conducted in the absence of any commercial or financial relationships that could be construed as a potential conflict of interest.

## Footnotes

1 Salamander is the common name for the order of Caudata, which includes newts and sirens.
