# Supplementary material for: Fostering Children’s Connection to Nature Through Authentic Situations: The Case of Saving Salamanders at School
Source: Front Psychol. 2018 Jun 8;9:928. doi: 10.3389/fpsyg.2018.00928 (PMC6002744; doi:10.3389/fpsyg.2018.00928)
Supplement: Supplementary file 1 [file Data_Sheet_1.DOCX]

**Appendix A**

**Summary of Field Observations 2015**

| Field Observation | Of what? | Date | Group | Which participation time? | How many Salamanders found? | Our role |
| --- | --- | --- | --- | --- | --- | --- |
| 1 | Salamander Project | 13 April | 4C, 1 | 1^st^ time | 70 | Observer |
| 2 | Intro lesson | 16 April | 4B | NA | NA | Observer |
| 3 | Salamander Project | 20 April | 4C, 1 | 2^nd^ time | 1 | Participant Observer |
| 4 | Salamander Project | 22 April | 4B, 2 | 1^st^ time | 1 | Participant Observer |
| 5 | Salamander Project | 24 April | 4A | 2^nd^ time | 2 | Participant Observer |
| 6 | Salamander Project | 29 April | 4B, 2 | 2^nd^ time | 4 | Participant Observer |
| 7 | Salamander Evening (community event) | 8 May | NA | NA | 1000 + (salamanders counted in pond) | Participant Observer |
| 8 | Salamander Project | 13 May | 4A, 1 | 4^th^ time | 9 | Participant Observer |
| 9 | Salamander Project | 25 May | 4C, 3 | 3^rd^ time | 0 | Participant Observer |
