# Supplementary material for: Fostering Children’s Connection to Nature Through Authentic Situations: The Case of Saving Salamanders at School
Source: Front Psychol. 2018 Jun 8;9:928. doi: 10.3389/fpsyg.2018.00928 (PMC6002744; doi:10.3389/fpsyg.2018.00928)
Supplement: Supplementary file 2 [file Data_Sheet_2.DOCX]

**Appendix B**

**Salamander Project Questionnaire 2015**

**Write 3 words that best characterise the Salamander Project for you:**

**Did you find any salamanders yourself?**

**How many? (if you can remember!)**

**Were you at the Salamander Evening?**

**Did it feel like you had missed out on your lunch break when you participated in the project?**

**Do you feel that you care more about salamanders now than before the project?**

**Write down a particular memory you have from the project:**
