# Supplementary material for: Fostering Children’s Connection to Nature Through Authentic Situations: The Case of Saving Salamanders at School
Source: Front Psychol. 2018 Jun 8;9:928. doi: 10.3389/fpsyg.2018.00928 (PMC6002744; doi:10.3389/fpsyg.2018.00928)
Supplement: Supplementary file 3 [file Data_Sheet_3.DOCX]

**Appendix C - Interviewees 2015 and 2017**

| **Participant** | **Class** | **Gender** | **Interviewed 2015** | **Interviewed 2017** |
| --- | --- | --- | --- | --- |
| 1 | 4a, | G | X |  |
| 2 | 4a, 6a | G | X | X |
| 3 | 4a, 6a | G | X | X |
| 4 | 4a, 6a | G | X | X |
| 5 | 4a | B | X |  |
| 6 | 4a, 6a | B | X | X |
| 7 | 4a | B | X |  |
| 8 | 4a | B | X |  |
| 9 | 4b, 6b | G | X | X |
| 10 | 4b | B | X |  |
| 11 | 4b | G | X |  |
| 12 | 4b | B | X |  |
| 13 | 4b | G | X |  |
| 14 | 4b, 6b | B | X | X |
| 15 | 4b | G | X |  |
| 16 | 4b, 6b | B | X | X |
| 17 | 4c | B | X |  |
| 18 | 4c, 6c | G | X | X |
| 19 | 4c, 6c | G | X | X |
| 20 | 4c | B | X |  |
| 21 | 4c, 6c | B | X | X |
| 22 | 4c | G | X |  |
| 23 | 4c, 6c | G | X | X |
| 24 | 4c | B | X |  |
| 25 | 4c | G | X |  |
| 26 | 6a | B |  | X |
| 27 | 6a | G |  | X |
| 28 | 6a | B |  | X |
| 29 | 6a | B |  | X |
| 30 | 6b | G |  | X |
| 31 | 6b | G |  | X |
| 32 | 6b | G |  | X |
| 33 | 6b | B |  | X |
| 34 | 6b | G |  | X |
| 35 | 6c | G |  | X |
| 36 | 6c | G |  | X |
| 37 | 6c | B |  | X |
| 38 | 6c | B |  | X |
| **Total 38** | **4a - 8**  **4b - 8**  **4c - 9**  **6a - 8**  **6b - 8**  **6c - 8** | **18 boys**  **20 girls** | **25** | **24**  **(Including 11 that were interviewed in 2015)** |
