# Supplementary material for: Fostering Children’s Connection to Nature Through Authentic Situations: The Case of Saving Salamanders at School
Source: Front Psychol. 2018 Jun 8;9:928. doi: 10.3389/fpsyg.2018.00928 (PMC6002744; doi:10.3389/fpsyg.2018.00928)
Supplement: Supplementary file 4 [file Data_Sheet_4.DOCX]

**Appendix D**

**Interview guide 2015**

1. What is the best thing about the Salamander Project for you?

2. Is there anything that you think is not good about the project?

3. Do you have a special memory or story from the salamander project you could tell me about?

4. Could you explain for me how it feels to ‘work’ with salamanders? (How does it feel to search for them/find one/hold one?)

5.How does it feel to be part of the project?

6. What was different between the first and the last time you participated in the project? (Did something feel different?)

7. Can you tell me about what you have learnt from the project?

8. Was it different to the way you learn things in the classroom? (How?)

9. In what way(s) have your feelings towards salamanders changed with the project? (if they have changed)

10. In what way(s) have your feelings towards your classmates changed with the project? (if they have changed)

11. Do you feel like you have changed a bit yourself? (In what way?)

12. If you could decide, would you rather that the municipality got rid of the paddling pool so that the salamanders wouldn’t get stuck there anymore or would you prefer things to continue as they are now (with your school saving the salamanders every year and keeping the paddling pool)? What would be the best solution in your eyes? (Why?)

**Extra questions that often came up:**

Did you find any salamanders yourself? How many?

Had you ever seen a salamander before you started this project?

Do you feel like you know more about salamanders now than most other people? (How does that feel?)
