# Supplementary material for: Fostering Children’s Connection to Nature Through Authentic Situations: The Case of Saving Salamanders at School
Source: Front Psychol. 2018 Jun 8;9:928. doi: 10.3389/fpsyg.2018.00928 (PMC6002744; doi:10.3389/fpsyg.2018.00928)
Supplement: Supplementary file 5 [file Data_Sheet_5.DOCX]

**Appendix E**

**SALAMANDER PROJECT QUESTIONNAIRE 2017**

**For you who saved salamanders in 4th grade (2015)**

**Name: ______________________________________ Class: _______________**

1. How much do you remember from the Salamander Project? (tick a box)

🗆 I remember most things

🗆 I remember some things

🗆 I don’t remember much

1. What do you remember best? (please explain)
2. Did you enjoy the Salamander Project? Explain why/why not.
3. Do you remember approximately how many salamanders you found?
4. Do you remember how many times you participated in the project (went to the park and searched for salamanders)?
5. Have your feelings for salamanders changed since you participated in the project? (in other words during the last 2 years). How?

____________________________________________________________________________________

____________________________________________________________________________________

____________________________________________________________________________________

1. During the last 2 years, since you participated in the project, have you ever (tick the boxes that match your answer):

🗆 talked to someone about salamanders or the Salamander Project?

🗆 thought about salamanders of the Salamander Project?

🗆 seen a salamander?

1. When you were in 5^th^ grade did you notice that the 4^th^ graders participated in the Salamander Project? If yes, how did you notice this?

____________________________________________________________________________________

____________________________________________________________________________________

____________________________________________________________________________________

1. Did you go to the Salamander evening last year? (2016)

🗆 Yes 🗆 No

1. Do you think that the Salamander Project is an important part of your school? Why/Why not?

_____________________________________________________________________________________

_____________________________________________________________________________________

_____________________________________________________________________________________

_____________________________________________________________________________________

_____________________________________________________________________________________

1. When you think back, did the Salamander Project change the way you think about, or see, nature? (nature = the environment and all the species of animals and plants) How?

_____________________________________________________________________________________

_____________________________________________________________________________________

_____________________________________________________________________________________

__________________________________________________________________________________________________________________________________________________________________________

1. On a scale from 1 to 10, how important is nature for you?

_____________________________________________________________________________________

Thanks for completing this questionnaire!
