# Supplementary material for: Fostering Children’s Connection to Nature Through Authentic Situations: The Case of Saving Salamanders at School
Source: Front Psychol. 2018 Jun 8;9:928. doi: 10.3389/fpsyg.2018.00928 (PMC6002744; doi:10.3389/fpsyg.2018.00928)
Supplement: Supplementary file 6 [file Data_Sheet_6.DOCX]

**Appendix F**

**Interview guide 2017**

*Memory*

What do you remember about the Salamander Project (SP)?

What is your strongest memory from that time?

Can you remember how it felt to be part of the SP? (Can you describe for me those feelings?)

*Learning*

Can you tell me what you learnt/discovered through the SP?

Can you remember some facts about salamanders? (Can you give me some examples?)

How do you think you learnt these things?

This is your last year at Olovslund School. When you are older and you think back about this school, what do you think you will remember from your time here? (Do you think you will remember the SP?)

*SP culture/continuation*

Does it ever occur that you talk to someone about the SP or about salamanders? (for example explaining what you did when you were involved) To Whom? Approximately how often has this occurred?

Have you seen any salamanders since you were part of the project? (Explain when/where)

Last year, when you were in 5^th^ grade, did you notice the 4^th^ grade students participate in the SP? (How did you notice this? Did you ever talk to them or hear about how many they found?)

Do you think that the SP is an important aspect of your school? Why/ why not?

*Personal change/feelings*

Have your feelings for salamanders changed with the project? (In what way?)

Do you still care about salamanders? Would you help one if you saw somebody trying to hurt it?

Do you think you changed at all with the SP? How?

Did participating in the SP change the way you feel or how you see other animals? How?

Did participating in the SP change the way you feel or how you see nature as a whole? How?
